# Safety, Tolerability and Efficacy of Dietary Supplementation with Concord Grape Juice in Gulf War Veterans with Gulf War Illness: A Phase I/IIA, Randomized, Double-Blind, Placebo-Controlled Trial

**DOI:** 10.3390/ijerph17103546

**Published:** 2020-05-19

**Authors:** Drew A. Helmer, William W. Van Doren, David R. Litke, Chin-Lin Tseng, Lap Ho, Omowunmi Osinubi, Giulio Maria Pasinetti

**Affiliations:** 1 War Related Illness and Injury Study Center, Veterans Affairs New Jersey Healthcare System, 385 Tremont Avenue, East Orange, NJ 07018, USA; William.VanDoren@va.gov (W.W.V.D.); David.Litke@va.gov (D.R.L.); Chin-lin.Tseng@va.gov (C.-L.T.); Omowunmi.Osinubi@va.gov (O.O.); 2 Center for Innovations in Quality, Effectiveness and Safety, Michael E. DeBakey VA Medical Center, 2002 Holcombe Blvd., Houston, TX 77030, USA; 3 Department of Rehabilitation Medicine, New York University School of Medicine, 550 1st Avenue, New York, NY 10016, USA; 4 Icahn School of Medicine at Mount Sinai, 1 Gustave L. Levy Place, New York, NY 10029, USA; Lap.Ho@mssm.edu; 5 Department of Environmental & Occupational Health, Rutgers University School of Public Health, 683 Hoes Lane West, Piscataway, NJ 08854, USA; 6 James J. Peters VA Medical Center, 130 W Kingsbridge Road, Bronx, NY 10468, USA

**Keywords:** Gulf War veterans, Gulf War illness, dietary polyphenols, concord grape juice, safety, tolerability, cognitive functioning, fatigue, veteran

## Abstract

Approximately 30 percent of U.S. veterans deployed during the Gulf War (1990–1991) have been diagnosed with Gulf War Illness (GWI), a chronic multi-symptom disorder without widely available specific treatments. We investigated whether the consumption of Concord grape juice (CGJ), rich in anti-inflammatory flavonoids, would be tolerated and safe in individuals with GWI and explored improvement in cognitive function and fatigue. Thirty-six veterans with GWI enrolled in a 24-week randomized, double-blind, Phase I/IIA clinical trial to explore safety, tolerability, and feasibility of 16 ounces daily of commercially available CGJ compared to placebo. Participants completed neurocognitive tests and self-reported surveys at baseline, 12 and 24 weeks. Thirty-one participants (86%) completed the study; no dropouts were related to side effects. Thirty participants (83%) documented ≥80% adherence. There were no statistically significant unadjusted differences between CGJ and placebo groups in change in efficacy measures from baseline to endpoint. We employed general linear regression models controlling for baseline differences between groups which indicated statistically significant improvement in the Halstead Category Test–Russell Revised Version (RCAT) at endpoint in the CGJ group compared to placebo (8.4 points, *p* = 0.04). Other measures of cognitive functioning did not indicate significant improvements in the adjusted analyses (*p*-values: 0.09–0.32), nor did the fatigue variable (*p* = 0.67). CGJ was safe and well-tolerated by veterans with GWI. Our data suggest high tolerability and potential benefit from CGJ in veterans with GWI and can be used to inform future studies of efficacy.

## 1. Introduction

The Gulf War (1990–1991) was fought between United States-led coalition forces from 35 nations and Iraq in response to the Iraqi invasion and annexation of Kuwait [1]. Although the ground combat was relatively brief and there were few combat-related casualties relative to most conflicts, epidemiologic studies soon confirmed the existence of a chronic illness, now referred to as Gulf War Illness (GWI), in veterans of the Gulf War [2,3]. Approximately 25–30 percent of the 700,000 American veterans deployed to the Gulf War have been affected by this chronic multi-symptom disorder including the respiratory, gastrointestinal, and neurological systems [4].

There is currently no widely available specific treatment for GWI and, therefore, an urgent need exists to develop novel interventions either to resolve the underlying pathophysiology or to alleviate the effects of GWI. As elements of chronic inflammation are found in GWI [5], the potent anti-inflammatory properties of flavonoids, a subclass of organic chemicals called polyphenols found in some plants, may yield beneficial therapeutic effects [6]. In a randomized, double-blind clinical trial Golomb et al. found evidence that supplementation with the endogenous lipophilic antioxidant Coenzyme Q10 may benefit symptoms of GWI [7]. Further evidence highlights the potential value of flavonoids to alleviate chronic fatigue and preserve cognitive function [8]. While additional investigations are needed to clarify the mechanisms by which dietary flavonoids may improve symptoms, this evidence suggests the potential value of dietary flavonoids for alleviating symptoms and improving function in veterans with GWI. Preclinical studies using a rodent model of GWI have associated mild inflammation in the hippocampal formation of the brain with cognitive dysfunction [9]. Flavonoid metabolites have demonstrated bioavailability in the brain providing a plausible mechanism by which dietary flavonoids may act in GWI to suppress central nervous system inflammation [10].

We conducted a randomized, double-blind, placebo-controlled Phase I/IIA clinical trial to explore safety, tolerability, and feasibility of dietary supplementation with a commercially available Concord grape juice (CGJ) over a 6-month period. We also explored the efficacy of CGJ in treating cognitive deficits and chronic fatigue in veterans with GWI.

## 2. Materials and Methods

### 2.1. Study Design

**Figure 1:**
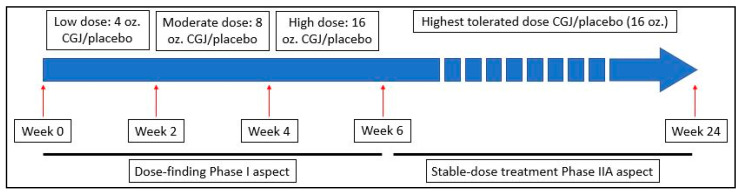
Timeline for the execution of the dose-finding and stable-dose treatment phases. CGJ—Concord Grape Juice.

This was a Phase I/IIA, 24-week, randomized, double-blind, placebo-controlled study in veterans with GWI conducted by a research team at the Icahn School of Medicine at Mount Sinai (ISMMS) in New York, NY and the Veterans Affairs New Jersey Health Care System’s (VANJHCS) War Related Illness and Injury Study Center (WRIISC) in East Orange, NJ. In the Phase I dose-escalation portion, subjects consumed a 4 oz. dose of study beverage (CGJ or placebo) daily for two weeks (weeks 0–2). Subjects were then instructed to increase consumption of study beverage to an 8 oz. daily dose for two weeks (weeks 3–4) and finally a 16 oz. daily dose (weeks 5–6). Subjects then immediately progressed into the Phase IIA steady-dose portion of the study where they remained in the same intervention arm consuming a 16 oz. daily dose for the remainder of the study (weeks 7–24). See Figure 1. All recruitment and data collection was performed by WRIISC personnel; complete assessments occurred at baseline, midpoint (week 12), and endpoint (week 24).

A random number table was generated (www.randomization.com) to randomize subjects into the CGJ or placebo arm upon enrollment, and blocks of four participants per block were used to ensure approximately equal assignment to both arms. The research protocol was approved by the VANJHCS and ISMMS Institutional Review Boards and the Department of Defense Human Research Protection Office. This study is registered on ClinicalTrials.gov under the identifier NCT02915237. All participants provided written informed consent prior to initiating research activities.

### 2.2. Treatment

The CGJ and placebo beverage were provided by Welch Foods, Inc., Concord, MA, USA. The CGJ was commercial grade 100% CGJ derived by hot press and pasteurized with no added ingredients. This juice has been utilized in previous studies and information concerning the specific polyphenol composition has been documented [11]. The placebo beverage was composed of high fructose corn syrup, fructose, corn syrup, natural grape essence, tartaric acid, malic acid, sodium citrate, red 40, blue 1, and blue 2, and was designed to match the CGJ with respect to color, taste, total calories, and sugar profile (ratio of glucose to fructose). The placebo contained no juice or polyphenolic compounds.

### 2.3. Participants

We proposed to enroll 60 Gulf War veterans aged 42–65 years who were suffering from GWI, as defined according to the Kansas Case Definition, for participation. This proposed sample size of 60 (30 CGJ and 30 placebo) was selected so that we would have 80% power to detect a 20% change in our endpoint efficacy outcomes and 92.6% statistical power to detect a 25% change. The Kansas Case Definition for GWI identifies 6 symptom domains (skin, pain, respiratory, neuro/cognitive/mood, gastrointestinal, fatigue) and requires endorsement of moderately severe and/or multiple symptoms in at least 3 of those domains. To meet the case definition, veterans also had to indicate that each symptom first became problematic during or after their Gulf War deployment (August 1990–July 1991) and not have any current medical conditions that may explain their symptoms (diabetes, cancer, among others specified) [12].

Individuals not meeting the previously defined criteria for Gulf War deployment and/or GWI were excluded. Exclusion criteria also included substance abuse within 6-months at the time of screening, unstable psychiatric conditions including suicidal or homicidal ideation, or schizophrenia. Additionally, individuals who reported consuming more than ten daily servings of foods/beverages rich in polyphenols at the time of initial phone screening were excluded. Participants who reported higher polyphenol intake after the initial screening were not removed from the study.

Recruitment was multifaceted. Flyers were posted at local healthcare facilities and distributed electronically on VA social media sites and Twitter. Deployed Gulf War veterans living within 100 miles of the study site were identified by the Department of Defense’s (DoD) Defense Manpower Data Center (DMDC) and mailed an information letter. If veterans did not opt out by phone or mail within two weeks from receipt of the letter, they were contacted by telephone. Gulf War veterans previously evaluated at the New Jersey WRIISC and who expressed interest in research were also contacted by letter and follow-up phone call.

At baseline, we characterized our sample by race, gender, smoking status, diet, age, body mass index (BMI), and overall symptom burden. All characterizations were derived from self-reported questionnaires. Overall symptom burden was captured using a variety of questionnaires including the Patient-Health Questionnaire-15 (PHQ-15), McGill Pain Questionnaire, Veterans Rand 36 Item Health Survey (VR-36), and Patient Health Questionnaire-8 (PHQ-8).

Participants’ overall diets were not tracked throughout the study, however, consumption of polyphenol-rich foods/beverages outside of the study intervention was captured using a Food Frequency Questionnaire (FFQ) administered at baseline, midpoint and endpoint. During interval check-ins (weeks 2, 4, 6, 8, 16, 20), participants were asked if their previously reported diets had changed; if so, the FFQ was re-administered. The FFQ consisted of a list of different food categories and asked participants to self-report how many servings of each item they consumed in a month (“Never or less than once per month”, “1–3 per week”, “Once a week”, “2–4 per week”, etc. up to “6+ per day”). High polyphenol-content foods/beverages were identified according to published approaches [13] and participants who consumed at least ten servings of polyphenol-rich foods/beverages per day were noted as high consumers.

### 2.4. Study Outcomes

Primary outcomes consisted of adherence, safety, and tolerability. Adherence to study beverage supplementation was determined through self-reported written and verbal logs. Written logs were collected at specified study timepoints (weeks 2, 4, 6, 8, 12, 16, 20, 24). If written logs were unavailable, verbal reports were communicated by subjects to study personnel and transcribed into written logs. Safety monitoring was conducted at all study timepoints using the PHQ-15 on which subjects rated the severity of each symptom from 0 (“not bothered at all”) to 2 (“bothered a lot”). New symptoms with an increase in severity of 2 from baseline were noted. Tolerability was monitored throughout the dose-escalation portion of the study during weekly telephone safety assessments that took place on weeks when other in-person or telephone survey assessments did not occur (weeks 1, 3, 5).

Secondary outcome measures included cognitive function and fatigue for evaluation of efficacy. Cognitive function was assessed by a neuropsychological battery (NPB) performed during baseline, midpoint (week 12), and endpoint (week 24). The NPB assessed domains of attention and response speed, memory, visuospatial functioning and executive functioning and included tests that, in published literature, were found to be the most sensitive for differentiating between veterans with and without GWI [14,15,16,17,18]. Measures sensitive to levels of inflammation [19] and the addition of polyphenols to a diet were also included [8,20]. See Table 1. Fatigue was evaluated using item “n” from the PHQ-15, “Since your last visit, how much have you been bothered by feeling tired or having low energy?” For safety monitoring purposes the PHQ-15 was administered at all study timepoints, however, analysis of the fatigue response was limited to baseline, midpoint, and endpoint sessions.

Adherence to beverage consumption was quantified as a proportion by summing the number of days the prescribed study beverage dose was consumed, divided by the number of days the subject was enrolled in the study. Adherence was calculated based on documented consumption; missing consumption logs were not considered in the calculation.

Tolerability to study beverage was quantified by summing the number of subjects that reached the highest daily dose of CGJ/placebo and maintained it throughout their participation and dividing by total study enrollment. Those subjects that dropped-out prior to reaching the maximum prescribed dose were calculated using the highest dose they received during participation before dropping out.

Safety monitoring of CGJ/placebo supplementation was quantified by summing the number of new symptom instances (when compared to reported symptoms at baseline) per evaluation timepoint. New symptom instances were also investigated for overlap between subjects to gauge any potential side-effects to CGJ/placebo.

Cognitive function was measured by converting raw scores obtained from the NPB to demographically corrected T-scores using the appropriate norm tables. Depending upon the test, we were able to correct scores for participant’s age, gender, ethnicity, and/or level of education. To assess the possible effect of the CGJ/placebo on cognitive functioning, measures of both the individual’s global cognitive functioning as well as their functioning in specific cognitive domains were included. When available, alternate forms of neuropsychological tests were used to minimize potential practice effects. Tests that are memory-based are generally more susceptible to practice effects [29], and consequently alternate forms are more likely to be published. Whenever available, alternate forms were used for follow-up assessments in this study (California Verbal Learning Test-Second Edition (CVLT-II), Brief Visuospatial Memory Test-Revised (BVMT-R)). Performance-based tests are generally less sensitive to learning effects and alternate forms are less likely to be published. Forms were reused in this study for Conner’s Continuous Performance Test-3 (CPT-3), Trails A & B, Wechsler Adult Intelligence Scale (WAIS-IV) subtests (Digit Span, Block Design), Stroop Color and Word test, and Halstead Category Test-Russell Revised Version (RCAT).

Three individual neuropsychological measures were selected *a priori* from the literature as most sensitive to cognitive dysfunction in veterans with GWI: the Stroop Color-Word test [executive functioning] [16], the CVLT-II [memory] [30], and the CPT-3 [attention and response speed] [31]. To capture overall cognitive performance, a composite Global Cognitive Functioning Score (GCFS) was created. Specifically, the 10 individual measures from the NPB were first grouped according to cognitive domain; then a cognitive domain score was calculated for each of the four domains by taking the mean of the demographically corrected T-scores for the specific tests in each cognitive domain. These four domain scores were then averaged to create a single GCFS score with equally weighted domain contributions. The four domains and the tests comprising them were: (1) attention/response speed—Trail Making Test Part A total time, CPT-3 detectability (d’), and Digit Span total recall (forward, backward and sequencing); (2) memory—BVMT-R total recall, CVLT-II immediate free recall total correct and CVLT-II List B free recall correct; (3) visuospatial—Block Design; and (4) executive—Trail Making Test Part B total time, Stroop Color-Word score, and RCAT total errors.

### 2.5. Statistical Analysis

We performed an intent to treat analysis. We evaluated whether group differences existed between CGJ and placebo at baseline, midpoint and endpoint and in the individual-level change from baseline to endpoint in the outcome variables using *t*-tests for two independent samples. We also employed general linear regression models to assess effects of group and time on the cognitive function and fatigue variables using a calculated individual-level difference between endpoint and baseline. We included baseline outcome values in the model as a covariate to address baseline imbalances. Observed power for the six selected efficacy outcomes were calculated.

## 3. Results

**Figure 2:**
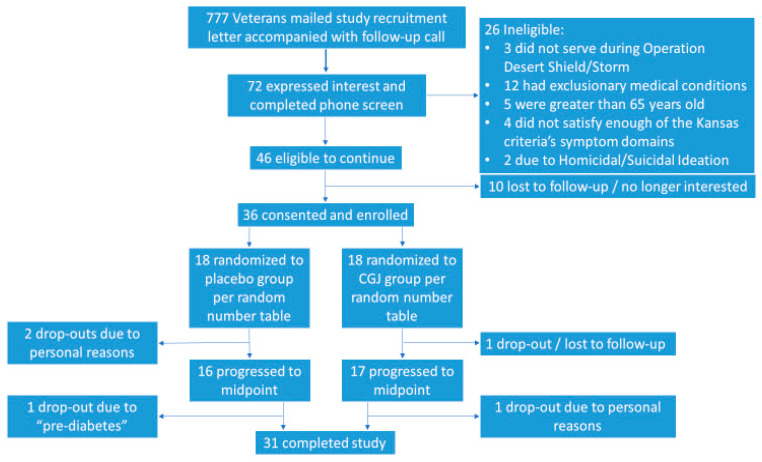
Subject selection and group assignment based on study design. CGJ—Concord grape juice.

We contacted 777 potential participants by letter and phone call. A total of 72 potential subjects were screened by phone for participation, 46 of whom were eligible to participate. Of those eligible, 36 participants were enrolled, with 18 being assigned to each the CGJ/placebo arms. Of the enrolled subjects, 33 continued to midpoint assessment and 31 completed the full 24-week protocol (Figure 2).

Participants were mostly men (81%) and Caucasian (58%) who all met Kansas criteria for GWI with symptom characteristics reflecting mild-moderate severity across the six domains. Mean body mass index was in the obese range (30.8 kg/m^2^, SD 4.9). There were no statistically significant differences between CGJ and placebo in these baseline characteristics.

Overall, participants’ measured cognitive function was in the normal range on the tests of the NPB, with only mean RCAT scores (41.3, SD 11.4) approaching clinical relevance with scores almost a standard deviation below the population normative mean of 50. See Table 2 for descriptive statistics of participants by randomization group. 

Adherence: 26 participants returned complete adherence logs, nine returned partial logs, and one returned no adherence data. Of those 26 veterans who returned complete logs, 25 had study beverage adherence equal to or greater than 80%. Using the partial data, five of nine subjects had study beverage adherence above the 80% threshold for the period with documentation. Considering both partial and complete adherence logs, 30 of our 36 subjects (83.3%) recorded an adherence rate equal to or above 80%.

Safety: A total of seven new symptoms were reported over the course of the entire study. When broken down by assessment encounter, week 20 had the highest number of new symptoms with three reported. When examined by group, differences emerged with the CGJ group contributing five of the new symptoms compared to two in the placebo group. Only one new symptom was reported by more than one participant; two participants reported a new problem in response to the item, “Since your last visit, how much have you been bothered by pain or problems during sexual intercourse?”

Tolerability: All participants increased daily dosage in accordance with the study protocol from 4 oz., to 8 oz., and finally 16 oz. No participants returned to lower dosage levels once target daily dosage was achieved.

Participant drop out was primarily due to unrelated personal issues (i.e., moved out of state, new work schedule that interfered with participation, death of a family member), with one complete loss to follow-up. One participant withdrew when their primary care provider indicated they were “pre-diabetic.” Upon review of their medical record, it was discovered they had elevated blood glucose levels in the non-diabetic range prior to enrollment in the study (diabetes was an exclusionary criterion, but not pre-diabetes). A higher proportion of women (three of seven (43%)) dropped out compared to men (two of 27 (7%)) and 3 of 11 (27%) African Americans dropped out compared to two of 25 (8%) Caucasians.

Efficacy: All potential measures of efficacy were considered secondary in this Phase I/IIa study. See Table 3 for descriptive statistics of measures of cognitive function and fatigue at each assessment.

Significant group differences on the GCFS (*p* = 0.03) and Stroop (*p* = 0.02) outcome variables were observed at baseline. Statistically significant differences between CGJ and placebo were also observed for Stroop at midpoint (*p* = 0.03) and endpoint (*p* = 0.02), GCFS at endpoint (*p* = 0.046), and RCAT at midpoint (*p* = 0.02) and endpoint (*p* = 0.01). Unadjusted change in mean demographically-corrected T-scores were not statistically significant for any efficacy measure.

Table 4 presents adjusted group contrasts on baseline-adjusted outcomes at endpoint, derived from general linear regressions. For most cognitive functioning variables (GCFS, Stroop (executive), CVLT-II (memory), and CPT-3 (attention & response speed)), there was no group difference in improvement (*p* values: 0.09–0.32). Results for the RCAT variable, representing the executive functioning domain, showed the CGJ group had greater improvement in cognitive functioning than the placebo group by 8.4 points (*p* = 0.04). There was no group difference for the fatigue variable (*p* = 0.67). Observed power ranged from 0.07 to 0.55 for the six efficacy outcome measures using the adjusted models.

## 4. Discussion

This Phase I/IIA, double-blind, randomized, placebo-controlled study clearly supports the safety and tolerability of CGJ at 16 ounces a day for 24 weeks in veterans with Gulf War Illness. In contrast, our secondary aim of exploring the efficacy of CGJ produced mixed results. The unadjusted differences between endpoint and baseline values were not statistically different between CGJ and placebo, however, general linear regression models controlling for observed baseline imbalances in the outcome measures demonstrated greater improvement in RCAT scores in the CGJ group compared to the placebo group at 24 weeks. No other efficacy outcomes demonstrated statistically significant group differences using the model to control for the baseline imbalances.

There were no clinically relevant, study-related adverse effects detected in this study; adherence was high and study withdrawal was almost universally due to personal reasons. It is not surprising that our study demonstrated the tolerability and safety of CGJ. A similarly designed study of older individuals with mild, age-related memory decline received up to 621 mL/day (roughly 21 oz.) CGJ or placebo for 16 weeks without any safety or tolerability concerns [20]. The CGJ utilized in our study is a commercially available food product with no known safety concerns. Our exclusion criteria were designed to mitigate the risk of elevated simple carbohydrate load in participants’ diets incident to treatment with CGJ by excluding individuals with a known diagnosis of diabetes. However, one participant voluntarily withdrew following notification of pre-existing pre-diabetes. In future studies of CGJ, it would be prudent to add pre-diabetes to the list of exclusionary criteria.

Our study was not powered to conclusively determine statistically significant group-specific changes in the efficacy measures. However, along with collecting information on our primary goals of evaluating the adherence, safety, and tolerability of CGJ, we collected potential efficacy measures at baseline, midpoint and endpoint to inform a future Phase II study of efficacy of CGJ or other flavonoid-rich preparations. We chose to focus on fatigue and objective measures of cognitive function as our efficacy measures based on pre-clinical studies [20,32]. We selected our battery of neuropsychological tests based on the experience and knowledge available at the time of study design about cognitive function in GWI and in trials of dietary polyphenols. We analyzed our results based on the *a priori* choices.

Of the cognitive tests assessed, individuals who consumed CGJ showed a significant improvement in the RCAT outcome, a measure of executive function, by study endpoint compared to placebo only after adjusting for the baseline values. The other efficacy outcome results were not statistically significant in either unadjusted or adjusted analysis and mixed in direction of association. CPT-3 captures attention and reaction time and may be affected by other factors present in our sample, such as comorbid PTSD, pain and fatigue. We were not able to disentangle these factors in our small sample.

With regard to the efficacy outcomes explored, our sample had near average performance on all neuropsychological tests despite reporting symptoms of cognitive dysfunction consistent with the Kansas criteria for Gulf War Illness. These findings indicate a disconnect between the participant’s experience of the cognitive symptoms and the objectively measured performance on structured assessments. It is possible that CGJ would have demonstrated a greater effect on objective cognitive performance in veterans with GWI with larger baseline deficits observed on testing. Future investigations of cognitive impact in GWI interventions could focus on veterans with GWI with more substantial measured cognitive deficits at baseline.

There have been few randomized controlled trials of interventions for Gulf War Illness and even fewer have used objective neuropsychological testing as outcome measures. Baraniuk et al. examined potential efficacy of L-carnosine [33] and found a possible cognitive benefit in a single neuropsychological test; the WAIS-R Digit symbol substitution test. The WAIS-R Digit symbol substitution test offers a practical and effective method to monitor cognitive function over time in clinical practice but has low specificity to determine which cognitive domain has been affected [34] making it somewhat comparable to the GCFS measure calculated for this report. The Baraniuk et al. study and this study both had imbalances in the cognitive efficacy measures at baseline between the intervention arms. Both studies also had mean standardized test scores within 1 standard deviation of the mean at baseline, reflecting modest objective cognitive deficit.

A study by Sullivan and colleagues found that veterans with high levels of exposure to pesticides and pyridostigmine bromide displayed stronger cognitive deficits than veterans exposed to a single toxin or low-level exposure to both toxins [35]. Thus, having a sample of veterans with unassessed, but likely heterogeneous, exposures may introduce variability in the underlying mechanism of the Gulf War Illness and the nature of the associated cognitive deficits. Future studies should consider controlling for or stratifying participants based on reported exposures to pesticides and other GW-associated toxins to the extent possible.

The measure of fatigue analyzed was a single self-reported item from the PHQ-15 with only three response levels. Our *a priori* measure of fatigue was the Chalder Fatigue Inventory which proved to be confusing to our participants and produced inconsistent reports; we abandoned analysis of this measure. Future studies should consider a more robust and validated measure of fatigue, such as the Multidimensional Fatigue Inventory (MFI) as recommended in the VA/DoD GWI Common Data Elements [36].

At baseline, both groups met Kansas criteria for Gulf War Illness. We assessed overall symptom burden in several ways: Patient-Health Questionnaire-15 (PHQ-15), McGill Pain Questionnaire, Veterans Rand 36 Item Health Survey (VR-36), and Patient Health Questionnaire-8 (PHQ-8). Overall, these metrics confirm the diverse and mild to moderate nature of the chronic symptoms reported by our sample. Other potential confounding characteristics, such as age, smoking status, BMI, and polyphenol intake also did not differ between groups.

Despite randomization, there were baseline imbalances in key outcome measures including Stroop and GCFS. Our statistical analysis adjusted for these differences. However, this may indicate other unmeasured differences, confounding our comparison. Future studies should consider stratifying randomization based on baseline performance on key outcome measures to avoid group imbalances as observed in this and other studies [33]. There is documented variability in cognitive dysfunction (and other symptoms) in veterans with GWI [17] and proactively mitigating baseline imbalances through study design could improve internal validity of randomized controlled trials. This problem could also be addressed with larger sample sizes or by focusing on certain types and intensities of GW exposures which have also been associated with differences in cognitive function [35].

The treatment duration of 24 weeks allowed sufficient time to ensure feasibility, tolerability and monitor for any short- and medium-term safety concerns. However, given the high symptom burden at baseline and the properties of the instrument used to assess symptom change, it is possible that our relatively low incidence of symptom adverse events was partially due to a ceiling effect. Future studies of GWI should consider utilizing symptom reporting instruments with a larger range of response outcomes.

By design, this is a small study with limited statistical power, further limited as we recruited only 36 of the targeted 60 participants (30 in each group) and retained only 31 at endpoint. Despite failing to achieve our target sample size, our findings support the safety, adherence, and tolerability of treatment with CGJ. Evaluation of secondary efficacy measures was hampered by low enrollment; we might have detected more definitive signals of efficacy had we reached our target sample size. Another limitation is our reliance on self-reported diet and adherence logs to monitor eating habits and adherence to the CGJ/placebo throughout the study. It is possible that variation in subjects’ dietary intake confounded our analysis. Potential participants who declined to participate often cited the burden of the in-person study visits, so we amended our protocol to reduce the number of in-person study visits from nine to three and, instead, conducted the assessments by telephone. This enhanced recruitment and retention but might have compromised the completeness and accuracy of our assessments of adherence. Finally, as can happen with many measures when given repeatedly over time, our study was subject to the practice effect regarding certain tests in the NPB. Although we used alternate forms when available during re-testing to minimize a practice effect, subjects still could have learned techniques to improve their scores over the three administrations.

## 5. Conclusions

Findings from this phase I/IIA, double-blind, placebo-controlled randomized clinical trial indicate that when administered at the studied dosage, CGJ was safe and well tolerated over a 24-week period, with no clinically relevant side effects. In secondary analyses of potential efficacy outcome measures, the RCAT, a measure of executive function, was significantly improved in the CGJ compared to placebo only when controlling for baseline group imbalances. The pilot data reported in this manuscript provide important preliminary information about candidate measures of efficacy and estimates of effect size for more definitive future studies.

## Figures and Tables

**Table 1:** Neuropsychological assessments by domain. Note: Three individual neuropsychological measures (bolded) were selected *a priori* from the literature as most sensitive to cognitive dysfunction in veterans with Gulf War Illness.

Attention & Response Speed	Memory	Visuospatial Functioning	Executive Functioning
Wechsler Adult Intelligence Scale (WAIS-IV): Digit Span Subtest [21]	**California Verbal Learning Test-Second Edition (CVLT-II)** [22]	WAIS-IV: Block Design Subtest [14]	**The Stroop Color and Word Test** [23]
**The Conner’s Continuous Performance Test-3 (CPT-3)** [24]	Brief Visuospatial Memory Test-Revised (BVMT-R) [25]		Halstead Category Test—Russell Revised Version (RCAT) [26]
The Trail Making Test A [27]			The Trail Making Test B [28]

**Table 2:** Key baseline characteristics by randomization group.

	Placebo (n = 18)		Concord Grape Juice (n = 18)		
	n (%)		n (%)		*p*-Value
African American (vs. Caucasian)	4 (22)		7 (39)		0.47
Women (vs. men)	5 (28)		2 (11)		0.42
Previous Smoker	6 (33)		8 (44)		0.51
Current Smoker	0 (0)		1 (6)		0.32
Never Smoker	12 (67)		9 (50)		0.32
High Polyphenol Consumers	8 (44)		10 (56)		0.52
	**Mean**	**SD**	**Mean**	**SD**	***p*-Value**
Age (years)	53.7	6.5	52.3	5.4	0.47
Body Mass Index (kg/m^2^)	30.9	5.3	30.7	4.7	0.92
Patient Health Questionnaire-15 Total Sum	15.1	5.4	15.1	5.9	1.00
Patient Health Questionnaire-15 Symptom Severity	1.5	0.2	1.5	0.3	0.70
Patient Health Questionnaire-15 Fatigue	1.8	0.4	1.6	0.6	0.22
McGill Sensory Pain Score	11.4	6.9	10.5	7.2	0.69
McGill Affective Pain Score	3.9	3.0	3.8	3.6	0.96
McGill Total Pain Score	15.3	8.3	14.3	10.1	0.75
McGill Average Intensity	1.8	0.5	1.7	0.7	0.86
Veterans Rand 36 Item Health Survey-General Health Score	40.8	22.9	41.7	20.5	0.91
Patient Health Questionnaire-8 Total Sum	13.8	5.8	12.1	7.0	0.44

**Table 3:** Mean demographically-corrected neuropsychological test (T scores) and fatigue scores across all timepoints by randomization group. (T-score range: 0–100, with higher values indicating better performance; PHQ-15 fatigue score range: 0–2, with higher values indicating more severe fatigue) CGJ—Concord grape juice; SD—standard deviation.

	Baseline					Midpoint					Endpoint					Δ Endpoint—Baseline				
	Placebo (n = 18)		CGJ (n = 18)			Placebo (n = 16)		CGJ (n = 17)			Placebo (n = 15)		CGJ (n = 16)			Placebo (n = 15)		CGJ (n = 16)		
	Mean	SD	Mean	SD	*p*	Mean	SD	Mean	SD	*p*	Mean	SD	Mean	SD	*p*	Mean	SD	Mean	SD	*p*
Global Cognitive Functioning Score (GCFS)	46.0	5.5	50.0	5.3	0.03	48.5	6.6	52.3	4.8	0.07	49.9	6.6	54.5	5.5	0.05	3.6	4.5	5.2	4.0	0.30
Stroop Color-Word Score	44.8	7.0	51.2	8.0	0.02	46.0	7.0	53.5	10.7	0.03	47.8	9.5	57.1	10.3	0.02	2.5	10.0	6.7	7.8	0.21
California Verbal Learning Test-Second Edition (CVLT-II) Immediate Free Recall Total Correct	52.8	13.0	56.3	8.0	0.33	50.4	10.4	56.5	9.3	0.09	57.7	11.3	61.3	8.1	0.32	3.7	8.9	6.0	7.4	0.45
Halstead Category Test—Russell Revised Version (RCAT) Total Errors	37.9	9.6	44.7	12.3	0.07	41.1	9.3	50.2	12.3	0.02	39.6	11.6	53.0	14.8	0.01	2.3	11.3	8.8	10.1	0.10
The Conner’s Continuous Performance Test-3 (CPT-3) Detectability (d’)	45.8	9.5	50.8	7.1	0.08	48.1	11.1	51.9	8.1	0.27	52.9	11.1	53.8	8.2	0.79	8.6	8.1	3.5	8.6	0.10
Patient Health Questionnaire-15 (PHQ-15) Fatigue	1.8	0.4	1.6	0.6	0.22	1.4	0.6	1.2	0.8	0.41	1.5	0.8	1.5	0.7	0.91	−0.3	0.9	−0.1	0.7	0.48

**Table 4:** Group contrasts in baseline-adjusted endpoint by outcome. Placebo group is the reference; positive values reflect greater improvement in cognitive functioning in the Concord grape juice group. Positive adjusted group contrast value for the fatigue variable represents more severe fatigue in the Concord grape juice group compared to placebo.

	Adjusted Group Contrast	Standard Error	*p*-Value
Global Cognitive Functioning Score (GCFS)	2.2	1.6	0.18
Stroop Color-Word Score	5.9	3.4	0.09
California Verbal Learning Test—Second Edition (CVLT-II) Immediate Free Recall Total Correct	2.7	2.7	0.32
Halstead Category Test—Russell Revised Version (RCAT) Total Errors	8.4	3.9	0.04
The Conner’s Continuous Performance Test-3 (CPT-3) Detectability (d’)	−3.2	3.1	0.31
Patient Health Questionnaire-15 (PHQ-15) Fatigue	0.1	0.3	0.67
